# Pre-operative granulocyte/lymphocyte ratio as a predictive marker of post-operative complications in patients with colorectal cancer

**DOI:** 10.3892/ol.2014.2669

**Published:** 2014-11-04

**Authors:** JIRO SHIMAZAKI, TAKANOBU TABUCHI, TAKESHI NAKACHI, GYO MOTOHASHI, KIYOTAKA NISHIDA, HIDEYUKI UBUKATA, TAKAFUMI TABUCHI

**Affiliations:** Department of Gastrointestinal Surgery, Ibaraki Medical Center, Tokyo Medical University, Ami, Ibaraki 300-0395, Japan

**Keywords:** granulocyte/lymphocyte ratio, colorectal cancer, post-operative complication, predictive marker

## Abstract

The aim of the present study was to assess the clinical relevance of the pre-operative granulocyte/lymphocyte (G/L) ratio as a predictive marker of post-operative complications in patients with colorectal cancer. In total, 85 patients (59 males and 26 females; mean age, 68.9 years) underwent surgery for colorectal cancer at the Department of Surgery, Ibraki Medical Center, Tokyo Medical University (Ami, Japan), and were divided into post-operative complication and non-complication groups. Clinical data, including age, gender, body mass index, tumor localization, tumor pathological type, cancer staging, surgery time, volume of surgical bleeding, pre-operative G/L ratio and further pre-operative laboratory data, including levels of albumin and C-reactive protein, Glasgow Prognostic Score, white blood cell count and levels of hemoglobin, creatine kinase, lactate dehydrogenase, carcinoembryonic antigen and carbohydrate antigen 19-9 were analyzed between these groups. The total post-operative complication rate was 18.8%. On univariate analysis, the amount of surgical bleeding and the pre-operative G/L ratio were significantly higher in the complication group than in the non-complication group (299.8±361.7 vs. 155.6±268.6 ml, P<0.05; and 6.73±10.38 vs. 3.49±2.78, P<0.05, respectively). Multivariate logistic regression analysis for the risk factors of post-operative complications, determined using univariate analysis, demonstrated that the amount of surgical bleeding and the pre-operative G/L ratio were independent risk factors of post-operative complications in patients with colorectal cancer. In conclusion, the G/L ratio may be a clinically relevant pre-operative predictive marker for post-operative complications.

## Introduction

As a result of significant improvements in surgical methods and techniques used in the treatment of colorectal cancer, the post-operative outcome of patients has improved. However, in certain cases, life-threatening complications may occur following colorectal surgery (1). The evaluation of the risk factors associated with post-operative complications in patients with colorectal cancer and the identification of prognostic indicators is required to reduce the post-operative mortality rate. Clinically, an increase in circulating leucocytes, primarily granulocytes, has been observed in patients with all advanced cancers; furthermore, the cellular immune reaction has been reported to decrease with disease progression in these patients (2). Several studies have demonstrated that the level of the granulocyte/lymphocyte (G/L) or neutrophil/lymphocyte (N/L) ratio may predict prognosis following surgery and/or chemotherapy in patients with colorectal cancer (3–8), however, no studies investigating the potential associations between the post-operative complications and pre-operative G/L ratio have been reported. Therefore, the current study reviewed our clinical experience with surgical therapy for colorectal cancer and retrospectively analyzed the clinical relevance of the pre-operative G/L ratio as a predictive marker of post-operative complications in patients with colorectal cancer.

## Materials and methods

Between January 2011 and June 2013, 85 patients (59 males and 26 females; mean age, 68.9 years) underwent surgery for colorectal cancer at the Department of Surgery, Ibaraki Medical Center, Tokyo Medical University (Ami, Japan). No patients had received pre-operative chemotherapy. The patients were divided into post-operative complication (n=16) and non-complication (n=69) groups. The patient medical records were reviewed and clinical data, including age, gender, body mass index (BMI), tumor localization, tumor pathological type, cancer staging, surgery time and volume of surgical bleeding were collected, in addition to pre-operative laboratory data, including levels of albumin and C-reactive protein (CRP), white blood cell (WBC) count, G/L ratio and levels of hemoglobin, creatine kinase (CK), lactate dehydrogenase (LDH), carcinoembryonic antigen (CEA) and carbohydrate antigen (CA) 19-9. The cancer staging was classified according to the degree of differentiation and the International Union Against Cancer tumor-node-metastasis classification (7th edition) (9). The Glasgow Prognostic Score (GPS) was calculated using the serum albumin and CRP levels (10). These 18 evaluative factors were compared between the groups. Written informed consent was obtained from all patients and the study was approved by the ethics committee of Ibaraki Medical Center, Tokyo Medical University (Ami, Japan).

Data are presented as the mean ± standard deviation (SD). Stat Mate IV (ATMS Co., Ltd., Tokyo, Japan) was used to perform the statistical analysis. The Mann-Whitney U test, Fisher’s exact test and the Pearson product-moment correlation coefficient were used to evaluate the correlations between variables in the univariate analysis, and a logistic regression model was used to perform multivariate analysis. P<0.05 was considered to indicate a statistically significant difference in all tests.

## Results

Of the 85 patients, 16 (18.8%) developed post-operative complications; six presented with an ileus (7.1%), five with an anastomotic leak (5.9%), two with surgical site infection (SSI; 2.4%), two with hepatic insufficiency (2.4%) and one with disseminated intravascular coagulation (DIC; 1.2%). In addition, two patients (2.4%) who developed hepatic insufficiency and DIC, respectively, succumbed to the disease within 60 days of the surgery.

Table I summarizes the clinical data for the complication and non-complication groups. The volume of surgical bleeding was significantly higher in the complication group (299.8±361.7 ml) compared with the non-complication group (155.6±268.6 ml; P<0.05). Although the surgery time was longer in the complication group compared with the non-complication group (199.8±75.4 vs. 180.2±81.4 min, respectively), the difference was not statistically significant. Furthermore, the differences in complications associated with age, gender, BMI, tumor localization, tumor pathological type and cancer staging were not statistically significant (Table I).

Table II reveals the comparison of the pre-operative laboratory data, including levels of albumin and CRP, WBC count, GPS, G/L ratio and levels of hemoglobin, CK, LDH, CEA, and CA19-9, between the complication and non-complication groups. The G/L ratio was significantly higher in the complication group compared with the non-complication group (6.73±10.38 vs. 3.49±2.78, respectively; P<0.05). However, no statistically significant difference was identified in the complications associated with differences in levels of pre-operative albumin and CRP, WBC count, GPS and levels of hemoglobin, CK, LDH, CEA and CA19-9 (Table II).

**Figure 1:**
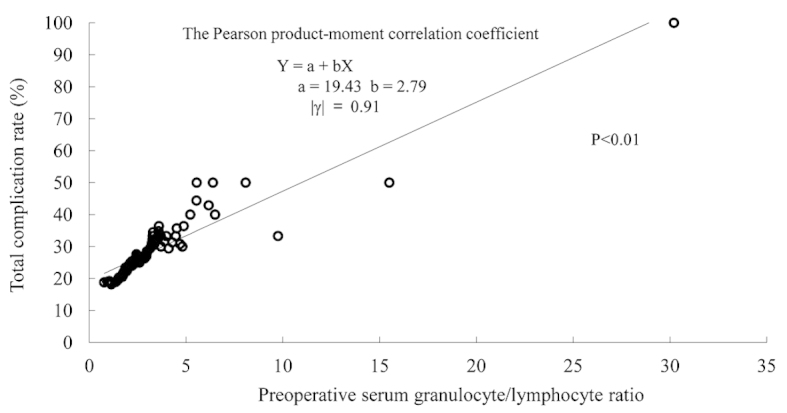
Linear regression established from the comparison between the pre-operative granulocyte/lymphocyte ratio levels and the total post-operative complication rate.

The multivariate logistic regression analysis, conducted using the complication risk factors determined by univariate analysis (the amount of surgical bleeding and the G/L ratio), revealed that the volume of surgical bleeding and the G/L ratio were independent risk factors of post-operative complications in patients with colorectal cancer (surgical bleeding: Odds ratio, 1.912; 95% confidence interval, 1.018–3.587; P=0.043; and G/L ratio: Odds ratio, 2.180; 95% confidence interval, 1.112–4.274; P=0.023; Table III). Fig. 1 shows the statistical regression line between the pre-operative G/L ratio levels and the total post-operative complication rate, with a statistically significant correlation (P<0.01; Fig. 1). The post-operative complication rate could be predicted from the pre-operative G/L ratio level.

## Discussion

Post-operative complications in colorectal cancer may result in functional impairments or even fatal outcomes for the patient, and also lead to an increase in medical costs associated with an extension of the hospital stay (11–13). Predicting the development of post-operative complications is useful in the perioperative management of patients, beginning pre-operatively and continuing into the post-operative period, and an early response to post-operative complications has contributed to a decrease in post-operative mortality rates (14,15).

The three most frequent post-operative complications of surgery for colorectal cancer are ileus, anastomotic leak and SSI. Chapuis *et al* (16) reported that the incidence of a post-operative ileus was 14.0% and that the risk factors included male gender, comorbidity with respiratory diseases, emergency surgery and if the surgery had been performed in >3 h. In addition, the incidence of anastomotic leaks and SSI have been reported to be 1.5–11.5% and 11.7–15.7%, respectively, with risk factors including high BMI, history of laparotomy, advanced-stage cancer and malnutrition (17–21). Yasunaga *et al* (22) reported that the incidence of complications in Japanese subjects following gastrointestinal surgery was at its lowest when the BMI was ~23.0 kg/m^2^, but that it was high when the BMI was ≤18.5 or ≥30.0 kg/m^2^. In the current study, the mean BMI of the subjects in the complication and non-complication groups was ~22.0 kg/m^2^, therefore indicating that there was no association between BMI and the development of post-operative complications. The nutritional assessment of patients with cancer is significant in improving the prognosis. Ishizuka *et al* (23,24) reported the GPS to be a post-operative prognostic factor in patients with colorectal cancer. However, in the current study, no association between the GPS and post-operative complications was identified. This is possibly due to the fact that the study was comprised of patients with a wide range of colorectal cancers, from early-stage to advanced-stage cancers with distant metastasis. A comparison between the complication group and non-complication group showed no statistically significant difference in GPS, as although the levels of CRP tended to be high in the complication group, there was almost no difference in the albumin levels. An albumin level of ≤3.5 g/dl is defined as low (and thus associated with increased risk) by GPS (10). In patients with colorectal cancer, problems associated with oral intake due to intestinal transit disorders, advanced-stage cancer or cachexia associated with distant metastases may result in hypoalbuminemia (25). Therefore, this indicates that if the control group was comprised of only patients with highly-advanced colorectal cancers, the GPS may be a risk factor for post-operative complications (26).

The present study examined 18 factors and focused on the pre-operative G/L ratio in the blood of patients with colorectal cancer, and further determined the correlation between the G/L ratio and post-operative complications. A correlation between the prognosis and the G/L or N/L ratios has been reported in various types of cancers (27–29). Clinically, patients with cancer occasionally present with an elevated WBC count, particularly an elevated granulocyte count, and a decreased lymphocyte count. When the progression and proliferation of the cancer is chronic and biologically invasive, it is accompanied by functional impairments due to localized tissue hemorrhage, ischemia or necrosis and causes various types of immune inflammatory responses. These responses include an elevated WBC count, particularly an elevated granulocyte count, the immunosuppression of lymphocytes, elevated levels of inflammatory cytokines, such as interleukin (IL)-6, and acute phase reactants, such as CRP, a negative nitrogen balance due to increased decomposition of muscle proteins, and the inhibition of albumin synthesis in the liver. The reactions are caused by a cytokine network composed of IL-6, IL-1 and tumor necrosis factor-α (30,31). IL-6 is a lectin, which is produced by T-cells, neutrophils, macrophages and tumor cells, and a cytokine that controls humoral immunity (32,33). The extent of biological invasion can be determined by the IL-6 levels; in colorectal cancers, IL-6 is reportedly associated with the progression of cancer and prognosis (34,35). We previously reported that a high G/L ratio prior to surgery is a risk factor associated with the prognosis of patients with colorectal cancer, and that a correlation exists between the G/L ratio prior to surgery and the IL-6 concentration (8,36). The findings of the present study suggest that a high G/L ratio prior to surgery is a risk factor for post-operative complications in patients with colorectal cancer. Taking the previous findings into consideration, a high G/L ratio prior to surgery in patients with colorectal cancer indicates high pre-operative IL-6 levels, i.e., a biological invasion, which is believed to cause post-operative complications. In addition, the present study also confirmed that the amount of surgical bleeding is a risk factor for post-operative complications. The volume of surgical bleeding reflects the damage to the biological tissue, which is accompanied by IL-6 production, and is considered to be strongly associated with post-operative complications (37,38).

Among the 18 factors that were examined in the present study, only the pre-operative G/L ratio and the volume of surgical bleeding were confirmed as risk factors for post-operative complications. Of these two factors, the G/L ratio allows for the pre-operative prediction of post-operative complications; furthermore, the G/L ratio is simple, easy to measure and is likely to aid in the future surgical management of patients with colorectal cancer. The findings of this study raise issues that remain to be discussed, particularly those pertaining to the types of post-operative complications for which the G/L ratio has specificity as a risk factor. To address these issues, further studies comprising a larger number of patients are required in the future.

In conclusion, to reduce the post-operative complications in patients with colorectal cancer, it is essential to reduce the possible amount of surgical bleeding. The pre-operative G/L ratio may be a clinically relevant pre-operative predictive marker in these patients.

## Figures and Tables

**Table I:** Clinical data between post-operative complication and non-complication groups.

Variable	Complication (n=16)	Non-complication (n=69)	P-value
Age, years	67.6±12.3	69.2±10.3	NS^a^
Male gender, n	13	46	NS^b^
BMI, kg/m^2^	21.9±4.7	22.5±3.9	NS^a^
Tumor localization, n			NS^b^
Colon	7	41	
Rectum	9	28	
Pathological tumor type, n			NS^b^
Well- to moderately-differentiated	16	64	
Poorly-differentiated	0	5	
Cancer staging, n			NS^a^
0–I	2	20	
II	4	23	
III	3	10	
IV	7	16	
Surgery time, min	199.8±75.4	180.2±81.4	NS^a^
Surgical bleeding, ml	299.8±361.7	155.6±268.6	<0.05^a^

a The Mann-Whitney U test and

b Fisher’s exact test were applied.

BMI, body mass index; NS, not significant.

**Table II:** Pre-operative laboratory data (mean ± standard deviation) between post-operative complication and non-complication groups.

Variable	Complication (n=16)	Non-complication (n=69)	P-value^a^
Albumin, g/dl	3.63±0.63	3.91±0.60	NS
CRP, mg/dl	2.42±3.28	1.15±3.02	NS
GPS	0.68±0.87	0.46±0.81	NS
WBC count, per μl	7143±1969	6462±1912	NS
G/L ratio	6.73±10.38	3.49±2.78	<0.05
Hemoglobin, g/dl	12.5±10.4	11.9±2.4	NS
CK, IU/l	94.0±89.2	90.9±71.2	NS
LDH, IU/l	270.5±178.7	227.8±164.9	NS
CEA, ng/ml	393.7±1089.8	111.3±464	NS
CA19-9, U/ml	165.6±527.6	627.2±2799.3	NS

a The Mann-Whitney U test was applied.

CRP, C-reactive protein; GPS, Glasgow Prognostic Score; WBC, white blood cell; G/L, granulocyte/lymphocyte; CK, creatine kinase; LDH, lactate dehydrogenase; CEA, carcinoembryonic antigen; CA, carbohydrate antigen; NS, not significant.

**Table III:** Multivariate analysis of the independent risk factors for post-operative complications.

Variables	Odds ratio	95% Confidence interval	P-value^a^
Surgical bleeding, ml (<100, 100–200, 200<)	1.912	1.018–3.587	0.043
G/L ratio (<2.5, 2.5–3.5, 3.5<)	2.180	1.112–4.274	0.023

a Multivariate logistic regression was applied.

G/L, granulocyte/lymphocyte.
